# Diphtheria

**Published:** 1864-05

**Authors:** John D. Jennings

**Affiliations:** Livingston, Ill.


ARTICLE XXI.

### DIPHTHERIA.

BY JOHN D. JENNINGS, M.D., of Livingston, Ill.

This is a disease of late origin; or, at least, we do not have any account of it as a distinct disease, prior to 1771. Dr. BARD, of New York, writing in that year, describes a false membrane, similar to that found in croup. In his description, he discards the notion of ulceration or gangrene, which was spoken of by previous authors on the subject of sore throat. Believing these ulcerations, when they do occur, to be complicated with other disease, or that they were mistaken, it being nothing but white exudation of coagulable lymph. We hear nothing more said about it until the year 1826, when BRETENEAU wrote a treatise on Diphtherite, in which he says it is not attended with ulceration or gangrene, but is closely identified in every respect with croup. Since his time, the disease has been described by many French writers.

This disease appeared in France, and prevailed as an epidemic in 1855–6, and 7, during which years a great number died. The recoveries were attended with great debility.

I now come to the epidemic that is described in this country during the last two years. It first attracted general attention in England, of late years, in the autumn of 1857 and summer of 1858—during which time it visited many towns; and, since then, has prevailed in very many parts. In this country there has been a very general prevalence of the disease, during the last two years, prevailing as an epidemic; and, in many localities, it has been very fatal. It is peculiar in its mode of attack, differing in almost every case but having, in the main, well marked characteristics that will distinguish it from other diseases, commencing usually with uneasiness in the fauces and pain on swallowing; gastric irritation, which frequently produces vomiting, disturbance of heptic functions, and a disturbance of the circulation. Sometimes the first symptoms are hoarseness, with altered tone of voice, not much swelling, but immediately an effusion of plastic lymph upon the mucous coat of the larynx and fauces, which becomes organized, spreading to the air passages, developing low fever, with a weak and quick pulse, hurried breathing, ending in death by asphyxia, the same as in pseudo-membranous croup, the majority of cases having a more insidious attack, with a more favorable termination. The first that attracts the patient's attention is pain on deglutition. On examining the throat, you find slight congestion, with considerable redness; here it may terminate, by acrid secretion relieving the congestion; but generally one or both tonsils become very much inflamed in a short time, sometimes with swelling of the parotid cervical and sub-maxillary glands; swallowing now becomes very painful; the tonsils and fauces have the appearance of indentation, looking like pits made by the kernels of wheat, showing that the mucous coat has become thickened in patches, which, with the effused lymph, gives it the appearance of ragged ulcers or gangrenous sloughs. This exudation is at first tough, elastic, white, or whitish-yellow shreds of membranes; but, as the disease progresses or becomes more malignant, it changes its color and becomes darker, with effusion of sanguino-purulent matter and fœtid breath, with the tonsils and fauces of a dark ash hue. This exudation, which is muco-sanguino fibrinous, spreads in the air passages of the head, giving a very offensive odor and increasing the danger of the patient, making convalescence very slow. Occasionally we have fever from the first symptoms of the disease, but not usually until the local inflammation of the throat has developed itself. The fever is of a low and malignant form; it may be sthenic in some, and is at the commencement, but does not remain so long, but soon assumes the æsthenic or typhoid character.

The blood is changed in its normal proportions to its relative constituents, having less fibrin and a tendency to disorganization; hence the frequent occurrence of epistaxis and sanguineous effusion. The nervous system is very deeply implicated, with general prostration of the whole system, which is the greatest danger of the disease. The stomach is often disturbed in the commencement, inducing emesis. Constipation of the bowels generally occurs, except when they are disturbed by the acrid secretions of the throat, which produces diarrhœa. During the inflammatory process in the throat, there is hypersecretion of the mucous coat, commingling with the lymph effused, thereby relieving the loaded vessels and preventing ulceration. The secretions are acrid in their character, producing excoriations upon any surface they come in contact with; the urine is acrid, scanty, and in the latter stages frequently entirely suppressed, followed by anasarca, and requires to be strictly attended to throughout the whole treatment of the disease. This is no doubt a blood disease, which develops its virulent poison upon the mucous coat of the throat and air passages, producing, in those predisposed, a pseudo-membraneous exudation; in others, inflammatory swellings of the tonsils and glands about the neck, depressing the vital as well as the nervous system. The virulence of the poison is such that it has a tendency to lessen the plasticity of the blood, by which its nutrition is destroyed, which is seen by not coagulating, becoming less in fibrin and deficient in red corpuscles, producing malignant æsthenic inflammations, depressing the whole system, causing the muscles to lose their tonicity, becoming totally and less cohesive, which may be accounted for in part by the poison inducing the disease, and in part by difficult respiration, thereby not relieving the blood of the waste material of the organized system by arterialization, which remains as poison; the febrile disturbance is generally slight; pain is not in proportion to other symptoms, sometimes quite absent; the voice dull and nasal, moderate thirst, and loss of appetite; it does not especially attack the puny and ill-fed, but is generally confined to those under 18 or 20 years of age. The appearance of the tonsils and fauces accords with the description given; bowels constipated; nervous system prostrated toward the close of the disease; secretions generally acrid; epistaxis with the blood not coagulable, but remaining watery, and in fact the solids and fluids in a septic condition. In all the malignant cases, the sweat and urine were decidedly acid; and, in fatal cases, the urine was entirely suppressed; in those dying from suffocation, by the false membrane, there was little or no swelling, not much fever, it being sympathetic or secondary. Dr. CLARK thinks it is contagious. During an epidemic, one thing I observed in the cases that I saw of that epidemic, and that was in nearly all cases of a malignant character, which lingered, or there was ulceration and gangrene, with sloughing of the fauces and other parts of the throat, and many patients were mortified before they died. Any external wound or abrasion on any surface was immediately followed by gangrene. The treatment will depend on our knowledge of the pathology of the disease. To counteract and eliminate the poison of the blood sustaining the patient, excite secretion and equalize the circulation are the main indications. In mild cases, little general treatment is required; the patient may take a dose of magnesia or some other saline cathartic, and use alum gargle. In somewhat severer cases, repeat the cathartic with ipecac and use a mixture of chlorate of potassa with lemon syrup, and, in addition to alum, use mur. tinct. ferri as a gargle; it has been recommended to use glycerine in false membranes as a solvent, which is good; but I think the chlorate of potash is better. You are debarred from blood-letting and tracheotomy on account of the nature of the disease producing ænemia in one case and gangrene in the other. When you have a high grade of inflammation, a full, strong pulse, with high fever, some would recommend bleeding; but it is found that venesection does not exercise the same controlling influence on inflammations of the mucous coat as other organs, and especially in this disease it does not relieve the tendency to plastic effusion; and again this disease early assumes the malignant type by a depraved state of the blood, which is shown by the dark hue and fœtid odor of the exudation; therefore it is better not to bleed and deplete too much. At first, you may give an active cathartic, and that one of a character that will stimulate the secretions, and at the same time not depress the vital powers. Calomel and ipecac, combined with carbonate of ammonia and soda, followed with Rochelle salts, if necessary, or any other saline cathartic, with the free use of chlorate of potassa, sesqui chloride of iron in tincture, diluted with nitrous ether. The preparation of potash I prefer, is—chlorate of potash, one ounce; syrup of lemon, four ounces; water, four ounces; sulphate of morphine, two grains. Dose, table spoonful every four or five hours; and use as a gargle the mur. tr. ferri, diluted with nitrous ether, also given two or three times a day, internally, as a tonic and a diuretic, together with soda and potash, which have a tendency to dissolve the coagulated lymph and keep the throat clean. It will be necessary to use expectorants, ipecac and opium, remembering to sustain the patient by iron spoken of, or quinine and ammonia with proper diet, without regard to inflammation; or, in other words, use constitutional treatment. You must counteract the poison in the blood by the potash, and eliminate from the system through the kidneys and pores of the skin by the above diuretics and diaphoretics. I do not think the free use of calomel in small doses beneficial, but the saline cathartics—soda, potash, and chlorates—should be freely used. For local treatment, use means to divert the blood from the inflamed parts and to equalize the circulation by sinapisms to the feet, legs, and hands or arms, external stimulants to the neck; and, as the disease advances, fomentations of hop poultices externally to the throat; use muriated tr. iron, nitrate of silver, sulphate of copper, alum, tanin or oak bark in advanced stages; apply sulphate of copper and nitrate of silver as a caustic, and, between caustics, use glycerine for its lubricating as well as its solvent powers. The lancet leeches and blisters, in malignant cases, should not be thought of; but, in their stead, use quinine, whine whey, carbonate ammonia, and animal broths, with fresh air, good nursing, and you may expect nine out of ten cases to recover.

